# Supplementary material for: pruR and PA0065 Genes Are Responsible for Decreasing Antibiotic Tolerance by Autoinducer Analog-1 (AIA-1) in Pseudomonas aeruginosa
Source: Antibiotics (Basel). 2022 Jun 6;11(6):773. doi: 10.3390/antibiotics11060773 (PMC9219673; doi:10.3390/antibiotics11060773)
Supplement: Supplementary file 1 [file antibiotics-11-00773-s001.zip › antibiotics-1746889-supplementary.pdf]

Table S1: Bacterial strains and plasmids.

Strain	Description	Reference
P. aeruginosa		
PAO1	Wild type	This study
PAO1-Tn5	Transposon (Tn-5) insertion mutant of PAO1	This study
Tn5-*pruR*	Tn-5 insertion mutant of PAO1, exhibiting transposon insertion on *pruR* gene	This study
Tn5-PA0065	Tn-5 insertion mutant of PAO1, exhibiting transposon insertion on PA0065 gene	This study
Δ*pruR*	Deletion mutant of PAO1, exhibiting deletion of *pruR* gene	This study
ΔPA0066-65-64	Deletion mutant of PAO1, exhibiting deletion of PA0066-65-64 operon	This study
Δ*pruR*/*pruR*	Complemented strain of *pruR* deletion mutant	This study
ΔPA0066-65-64/PA0066-65-64	Complemented strain of PA0066-65-64 deletion mutant	This study
Δ*pruR*/VC	Vector control of *pruR* deletion mutant, inserted with pJN105 vector	This study
ΔPA0066-65-64/VC	Vector control of PA0066-65-64 deletion mutant, inserted with pJN105 vector	This study
PAO1/VC	PAO1 inserted with pJN105 vector	This study
E. coli		
S17-1	*thi pro hsdR recA* RP4-2 (Tc::Mu) (Km::Tn7) λ-*pir*	[1]
JM109	*endA1 supE44 thi-1* Δ(*lac-proAB*) e14$^-$(*McrA*$^-$) F$^-$ [*traD36 proAB lacIqZ*ΔM15] λ-*pir*	[1]
XL2-Blue	Δ(*mcrA*)183 Δ(*mcrCB-hsdSMR-mrr*)173 *endA1 supE44 thi-1 recA1 gyrA96 relA1 lac* [*F proAB lacIqZΔ*M15 Tn10 (*Tetr*) *Amy Camr*]	Agilent Technologies
NEB 5α	*fhuA2* Δ (*argF-lacZ*) U169 *phoA glnV44* Φ80 Δ (*lacZ*) M15 *gyrA96 recA1 relA1 endA1 thi-1 hsdR17*	New England Biolabs
Plasmids		
pUT-miniTn5pro	pUT with a mini transposon containing *aacC1* Gm^R, R6K_γ origin, the *araC* gene, and an outward-reading ara_{BAD} promoter	[2]
pEX18Gm	Broad-host-range replacement vector; *sacB*, Gm^R	[3]
pJN105	Arabinose-inducible gene expression vector; pBRR-1 MCS; *araC-PBAD*; Gm^R	[4]

Table S2: Primers used in this study.

Primer	Sequence (5' to 3')
Transposon insertion site	
Para2	CATTGATTATTTGCACGGCGT
R6K	AGTACGTTAAACATGAGAGC
Deletion mutant construction	
pruR-1f	CGACTCTAGAGGATCCATGGTGCACCCGGACCA
pruR-1r	GGATGGCGCTCAGCCGAACATGAACGGCAACTGC
pruR-2f	GGCTGAGCGCCATCCGCC
pruR-2r	CCATGATTACGAATTACGGCAACTTCGACCTGG
PA0066-65-64-1f	CGACTCTAGAGGATCCAGGCGGAAGTCGCGCAG
PA0066-65-64-1r	GACGCCTGCTCAGGCGGTCACGATAGTTTTCCTC
PA0066-65-64-2f	GCCTGAGCAGGCGTCAGG
PA0066-65-64-2r	CCATGATTACGAATTCCTGCGCGACGACACCGC
pEX18Gm-s	TCAGGCTGAAAATCTTCTC
pEX18Gm-a	TAGCTCACTCATTAGGCA
pruR-os	GGGTCTTCGGATAGTCCACCTC
pruR-oa	GCAACGAGGAAGGCGAAGTG
PA0066-65-64-os	GTCGGTGACATGCTTGGTCC
PA0066-65-64-oa	GATCCGATCCTCAGCAGCATG
Complemented strain	
pJNSD*pruR*-1s	CGGGGGATCCACTAGAGGAAACAGCTATGTTCGA CACCCGCCTC
pJNSD*pruR*-1a	TATAGGGCGAATTGGTCAGCCCTTGCGCAGCGA
pJNSDPA0066-65-64-1s	CGGGGGATCCACTAGAGGAAACAGCTGTGACCAT TCGTTCCTACC
pJNSDPA0066-65-64-1a	TATAGGGCGAATTGGTCAGGCGGCGAGCTGGTC
pJN105-s	ATTAGCGGATCCTACCTG
pJN105-a	GGCGATTAAGTTGGGTAA
qRT-PCR	
pruR-fw2	ACCCGCCTCATCCGTCT
pruR-rv2	CCAGTTGGTGATGCTCGTG
PA0066-fw	GACATGCACCGCATCCG
PA0066-rv	GCACGCTGCCGTCCTG
rpsL-s	GCAACTATCAACCAGCTGGTG
rpsL-a	GCTGTGCTCTTGCAGGTTGTG
rpoS-F	CACTTCCTTCTCTTCCAAACAACA
rpoS-R	AGCTGCGTTGCGTCCAA

References

1. Murakami K, Ono T, Noma Y, Minase I, Amoh T, Irie Y, Hirota K, Miyake Y. Explorative gene analysis of antibiotic tolerance-related genes in adherent and biofilm cells of *Pseudomonas aeruginosa*. *J Infect Chemother* 2017; 23: 271-7. DOI: 10.1016/j.jiac.2017.01.004.
2. Siehnel R, Traxler B, An DD, Parsek MR, Schaefer AL, Singh PK. A unique regulator controls the activation threshold of quorum-regulated genes in *Pseudomonas aeruginosa*. *Proc Natl Acad Sci U S A* 2010; 107(17): 7916-21. DOI: 10.1073/pnas.0908511107.
3. Hoang TT, Karkhoff-Schweizer RR, Kutchma AJ, Schweizer HP. A broad-host-range *Flp-FRT* recombination system for site-specific excision of chromosomally-located DNA sequences: application for isolation of unmarked *Pseudomonas aeruginosa* mutants. *Gene* 1998; 212(1): 77-86. DOI: 10.1016/s0378-1119(98)00130-9.

4. Newman JR and Fuqua C. Broad-host-range expression vectors that carry the L-Arabinose-Inducible *Escherichia coli araBAD* promoter and the *araC* regulator. *Gene* 1999; 227(2): 197-203. DOI: 10.1016/s0378-1119(98)00601-5.
